# Impact of Different Binders on the Roughness, Adhesion Strength, and Other Properties of Mortars with Expanded Cork

**DOI:** 10.3390/ma11030364

**Published:** 2018-03-01

**Authors:** Danuta Barnat-Hunek, Marcin K. Widomski, Małgorzata Szafraniec, Grzegorz Łagód

**Affiliations:** 1 Faculty of Civil Engineering and Architecture, Lublin University of Technology, Nadbystrzycka St. 40, 20-618 Lublin, Poland; malgosiaszafraniec@gmail.com; 2 Faculty of Environmental Engineering, Lublin University of Technology, Nadbystrzycka St. 40B, 20-618 Lublin, Poland; g.lagod@pollub.pl

**Keywords:** heat-insulating mortar, expanded cork, roughness, adhesion

## Abstract

The aim of the research that is presented in this paper was to evaluate the physical and mechanical properties of heat-insulating mortars with expanded cork aggregates and different binders. In this work, the measurements of surface roughness and adhesion strength, supported by determination of basic mechanical and physical parameters, such as density, bulk density, open porosity, total porosity, absorbability, thermal conductivity coefficient, compressive strength, flexural strength, and frost resistance of mortars containing expanded oak cork, were performed. The scanning electron microscope (SEM) investigations demonstrated the microstructure, contact zone, and distribution of pores in the heat-insulating mortars containing expanded cork. The results indicated that the addition of expanded cork and different binders in heat-insulating mortars triggers changes in their roughness and adhesion strength. The SEM research confirmed the very good adhesion of the paste to the cork aggregate.

## 1. Introduction

Cork materials are made from bark, i.e., the external dead layer of the cork oak (*Quercus suber*). This tree grows in the Mediterranean region, especially in its western part: Portugal, Spain, South France, Italy, and Croatia, and in North Africa—Tunisia, Algeria, and Morocco [1,2]. The biggest producer of cork worldwide is Portugal, which provides more than half of the world’s cork demand. *Quercus suber* has a thick layer of cork, which is homogeneous and regular in its structure. This growing covering tissue used for cork production is composed of microcells, the shape of which resembles a tetrakaidecahedron connected by capillaries. Therefore, cork has very good physical and mechanical properties, such as good thermal conductivity and acoustic insulation, vibration resistance, and high durability. In 1 cm^3^ of cork mass, there are as many as 40 million tetrakaidecahedral microcells. Suberin (45%), lignin (27%), cellulose and polysaccharides (13%), tannins (6%), wax (5%), and others (5%) are the main structural components of cork [3].

Cork is characterized by low specific weight equal to 190–250 kg∙m^−3^ due to the gas mixture filling its microcells in 90%. Moreover, taking into account the low water absorption reaching approximately 18–20%, cork can be considered almost unsinkable. Cork has a low thermal conductivity coefficient λ equal to 0.037–0.040 W∙m^−1^∙K^−1^ and a very high specific heat capacity. This makes cork a better material in comparison to, for example, extruded polystyrene, because it can work in a higher temperature range, which does not affect the insulation properties, as in the case of Styrofoam, which can evaporate. Cork absorbs up to 70% of sound waves. The high thermal inertia related to a huge value of specific heat capacity makes cork a flame resistant material. Additionally, cork has low water absorption (up to 20%) and impermeability to liquids and gases [4,5].

In building industry, the properties of cork are used in thermal, acoustic, and vibration insulation [1]. Moreover, cork exhibits considerable strength and durability. Therefore, its main application focuses on utilizing the above-mentioned properties. Granulated cork is used as a filler of wall and roof voids, primarily for thermal insulation. Additionally, granulated cork is a perfect underlay for pitches with artificial grass. Expanded cork is a specific type of cork aggregate. Its distinctness results from the manufacturing process, based on cork exposition to a high temperature. Cork granules expand and are bound by suberin, a natural binder activated at high temperature. This material is fully natural and ecological because it contains no artificial binder.

Following the trend of energy-efficient construction, building mortars nowadays undergo modifications to achieve greater thermal insulation in order to reduce heat emission from buildings [6]. Lightweight aggregates are added to mortars in order to improve their thermal properties, reduce the effects of water condensation, and minimize the transfer of heat through thermal bridges [3], the impact of which is significantly visible, especially in Eastern and Northern Europe [7]. Traditional mortars are being nowadays replaced by lightweight heat-insulating mortars [3,6,7]. In heat-insulating mortars, the amount of a typical aggregate such as sand is reduced and replaced by lightweight aggregates [3,4,8,9,10,11,12]. Investigations on lightweight heat-insulating mortar with recycled expanded cork are shown in the paper by Moreira, António, and Tadeu [4]. It was found that replacing sand with expanded cork decreased the density, compressive strength, and thermal conductivity of the mortar [1,12]. The lower amount of cement in the mortar decreased the value of its thermal conductivity coefficient. The mixtures containing the lightweight aggregate, such as cork, had a higher coefficient of vapor permeability than the reference samples. Thus, these mortars were more effective in preventing unwanted internal condensation of water vapor [4]. Research reported by Bras, Leal, and Faria [3], when compared traditional mortars with those comprising granulated cork or extruded polystyrene. An analysis of mixtures in which sand was replaced by mass with cork in a volume from 0 to 80% was carried out in their study. The results were similar to those shown in the paper by Moreira et al. [4], and the experiment confirmed the conclusions formulated in the previously discussed paper. In the reported case of mortars with granulated cork, an increased dose of cork led to a linear decrease in thermal conductivity, which was also associated with a decrease in density [4]. Mortars with granulated cork were considerably more stable and less prone to temperature changes per kilogram of weight in unstable conditions [3]. A different correlation for granulated cork was described, in which the absorption value decreased significantly in the phase of replacing sand with 20–50% of cork. In the case of 50–70% aggregate replacement, the value of this coefficient was much smaller than in the case of mortars with extruded polystyrene, which may positively influence the moisture conditions inside buildings [3]. The use of cork in concrete production means an almost total loss of concrete mechanical properties, which was proved by Branco et al. [5] and Guerra et al. [9]. Branco et al. also showed that, when expanded cork was used to replace the aggregates, the strength loss was generally higher than in the case of natural cork application. A 61.1% decrease in compressive strength was observed when sand was replaced by 30% of expanded cork. In turn, in the case of application of natural cork, the compressive strength was decreased by 74.5% [5]. Guerra et al. reported that cork powder concrete exhibited from 4- to 6-fold lower indirect tensile strength than the control concrete and from 12- to 20-fold lower compressive strength than the control concrete [9].

The relatively low density of cork is related mainly to the high gas content inside the small cells, typically with a length of 40 mm. The very poor heat transfer properties of cork are a result of both the gas content and cell size. In the case of cork, only conduction has significant importance for heat transfer. However, the thermal conductivity of the walls equal to 0.045 W∙m^−1^∙K^−1^ is only approx. twice higher than the conductivity of the gas inside the cells, 0.02 W∙m^−1^∙K^−1^ [13]. In the research carried out by Karade et al. [14], the reduction of compressive strength of cement-cork blends was observed. It was found that the thermal conductivity of concrete-cork composites decreased as the concrete density dropped. For concrete containing 20% cork, 46% greater thermal resistance was measured in comparison to concrete without additions [10]. The aim of another research [11] was to evaluate the mechanical properties of cork-based agglomerates, which may be used as sandwich panel components for lightweight structures. In papers [15,16,17,18,19,20], studies on the fire resistance of composite panels were reported. It is a serious issue related to practical application of lightweight ecological fillers, i.e., fire-resistant materials based on e.g., cork-based composites [16]. A significant amount of information related to the fire performance of polymers, mainly on their thermal decomposition in fire, fire damage to composites, and fire resistance of polymers with applied sand fire retardants, was presented by Mouritz and Gibson [20].

The aim of the paper [12] was to show the influence of hydrophobization on samples of heat-insulating mortars with expanded cork. Thin film silicon samples were secured against water infiltration into the samples and were protected from ice crystallization. It was also shown [21] that the products that were made from cork accumulated and stored carbon for long periods. Papers [22,23] presented a study on application of Life Cycle Assessment (LCA) for evaluation of the environmental impacts of the raw cork production. Cork is being predominantly used as a raw material in the most profitable cork stopper production [22]. Important differences were observed in environmental studies between various types of production, mainly due to the intensity and repetition of forest activities [23]. Bowyer [24] performed LCA studies for cork products used as construction materials such as flooring. These studies showed some advantages of cork-based mortars in comparison to traditional mortars and mortars with extruded polystyrene [24]. There is no doubt that they have a positive influence on the indoor environment.

The interfacial roughness of mortar plays a significant role in surface bonding to building materials. To the best of our knowledge, heat-insulating mortars containing expanded cork were not tested for their roughness. Thus, it seems necessary to determine the quantitative relationship between the mechanical interlock and the interfacial roughness of mortars because the quality of surface and the mortar roughness greatly influence the type of failure [25]. The parameters of mortars roughness can be also used to predict the strength and durability of bonds between the mineral base and the mortar.

Our research was focused on the influence of surface roughness of mortars containing cork on their adhesion strength and the other strength characteristics. The literature studies showed that the effects of the surface roughness of mortars containing expanded cork, different in relation to the applied binder and fine-grain aggregate, on the strength of adhesion to the brick surface and the other strength characteristics are unknown. The studies of the cork-based mortar surface were aimed at possible indication of the diversified geometrical structure of the tested mortars, with a view to the aspect of their mechanical adhesion to the surface. An additional aim of the research that is presented in this paper was to evaluate the physical and mechanical properties of heat-insulating mortars with expanded cork containing different binders.

The compositions of the tested mortars were deliberately designed not to focus on the impact of the varying percentages of the cork, because it was often tested and is basically obvious [1,3,4,7,14]. The novelty of the presented research, in addition to the presentation of the relation between the surface roughness and adhesion of mortars, was also based on demonstration of the observed changes in previously described properties of mortars containing expanded cork and different amounts of binders (cement, lime) and containing, or not, fine-grained aggregates, such as sand. Thus, no control mixture in our research was designed because all of the tested mortars contained expanded cork. In our opinion, the mixtures between cork and cement/lime/sand, and particularly the change in their microstructure, are an important topic, which needs to be developed and studied concerning not only the strength, thermal properties, and durability, but also the surface roughness and adhesion of mortars.

## 2. Materials and Methods

Four mixtures of the expanded cork mortar were prepared. The compositions of the mortars with the expanded cork aggregate are presented as volumetric ratios in Table 1. As shown in Table 1, in most of the studied cases, the cork content in the tested mortars was the same and the applied compositions of the mortars varied in the content of binders (cement and lime) and/or fine-grained aggregates (sand). Only in one case, the applied cork content was changed to allow at least partial comparison of the selected properties of different mortars in relation to the presence of cork.

The hydrated lime complied with the requirements of PN-EN 459-1:2015-06 [26] and was characterized by an apparent density of 390–410 kg∙m^−3^. The composition of the lime, in terms of oxides, was as follows: CaO—95.5%, CO_2_—2.1%, MgO—0.5%, SO_3_—0.1%, and free water—1.5%. The CEM I 32.5R was prepared according to the Polish standards PN EN 197-1:2012 [27] and PN-B-19707:2013-10 [28].

The technical parameters of the applied Portland cement CEM I 32.5R are presented in Table 2.

The chemical composition of the quartz sand is shown in Table 3.

**Figure 1:**
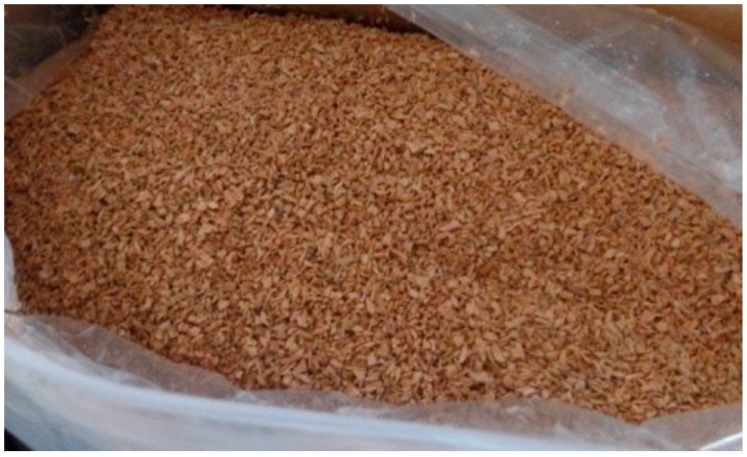
Natural granulated cork with a grain size of 1–2 mm.

Mortars were developed by adding expanded cork with a grain size of 0.5–1 mm and 1–2 mm (Figure 1). The characteristics of the applied expanded cork are presented in Table 4.

An ethylene vinyl acetate copolymer at a level of 0.15% was used in mortars M1, M2, and M4 as a plasticizing-reinforcing admixture. The polymer was added to the analyzed mortars to improve their workability and mixture flexibility, increase water retention in the binder, reduce the rate of evaporation, and to significantly improve the adhesion to all of the construction substrates. A special kind of redispersion powder containing a hydrophobic additive was used for the studies, which yielded composites with higher resistance to water. The main goal of the applied hydrophobization was to increase the limit of the surface tension between water and the impregnated material so that the difference should be as high as possible [8,29], according to suggestions presented by Frattolillo et al. [30] and Formia et al. [31].

**Figure 2:**
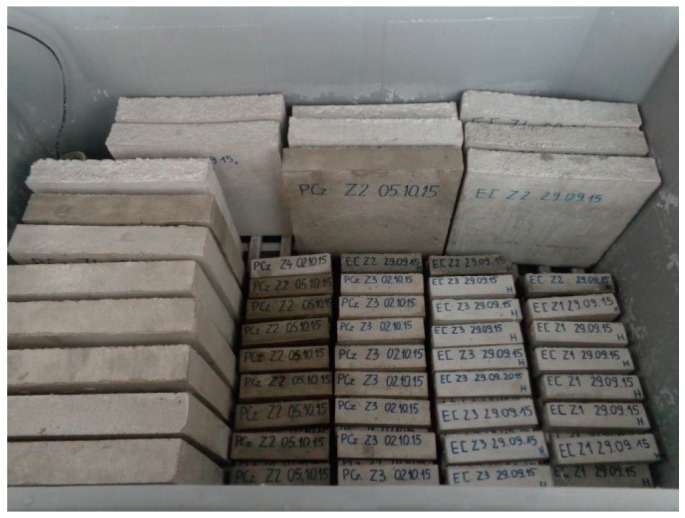
Demolded mortar samples.

Samples with the dimensions of 40 × 40 × 160 mm were prepared based on the EN 196-1:2016-07 standard [32]. The mixture was first mixed for 4 min in a mixer and then placed in a mold in two layers. Both of the layers were properly compacted for 1 min on a jolting table and the top layer was aligned. The samples were cured under standard conditions for 24 h and then demolded and stored in a climatic chamber at 23.5 °C and relative humidity of 73.5% for 21 days. In turn, 300 × 300 × 50 mm samples were used to test the thermal conductivity of the studied mortars. Due to the significantly greater volume of samples designed to test thermal conductivity, influencing their greater moisture and prolonged time of bonding, the samples were demolded after 72 h (not after 24 h as in the case of previously described measurements) and stored in a climatic chamber with the other samples (Figure 2). After 24 h, the samples were too wet to allow successful demolding; hence, the prolonged time of molding.

Six samples from each mortar mixture were used for the measurements of density, porosity, compressive strength, absorbability, and frost resistance, whereas the measurements of flexural strength and thermal conductivity involved three samples from each of the mortar mixture.

**Figure 3:**
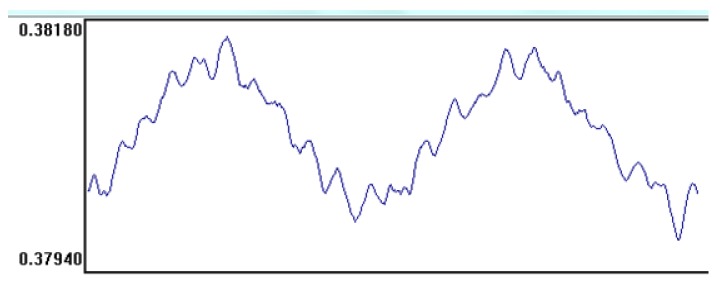
Stabilized graph of the λ coefficient for mortar M4.

Determination of bulk density, density, and total porosity was performed, according to the PN-EN 1015-10:2001 standard [33]. A mortar absorptivity test was carried out according to standard BS 1881-122, 2011 [34]. The samples were dried to constant dry mass before measurements at a temperature of 60 °C ± 5 °C in a laboratory oven. The absorptivity test was performed under laboratory conditions at an ambient temperature of 20 ± 2 °C. A plate apparatus FOX 314, TA Instruments, New Castle, DE, USA with 300 × 300 × 50 mm plates was used to determine the thermal conductivity coefficient λ. The tested samples were dried until they reached a constant weight. In order to determine the thermal conductivity coefficient of the mortar, two values of temperature were applied: 20 °C for the heating plate and 0 °C for the cooling plate. The average temperature was 10 °C. The measurements were based on allowing a specific heat flow through the sample and determination of temperatures measured for a given heat flow on the inflow and outflow surfaces. The device that was used for the λ coefficient test cooperated with a computer and WinTherm32v3 software by LaserComp, Saugus, MA, USA, in which the test results were registered. Figure 3 shows an example of a graph prepared for one of the tested mortars with WinTherm32v3 software.

**Figure 4:**
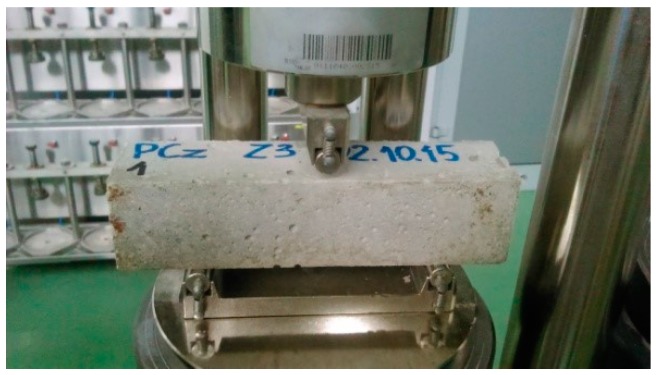
Mortar sample in the flexural strength-testing machine.

**Figure 5:**
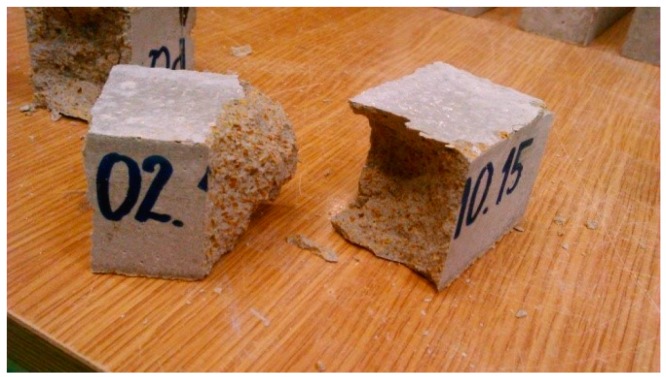
Samples of heat-insulating mortar after the compressive strength test.

The flexural strength of the mortars was determined according to the EN 1015-11:2001 standard [35]. Rectangular prisms of each mortar with the dimensions of 40 × 40 × 160 mm were used (Figure 4). The tests were performed after 21 days of sample curing. The samples were loaded with a centrally applied force (3-point bending). The load increase was set at 20 N∙s^−1^. This experiment involved pieces of test specimens obtained just after the flexural strength test. The compressive strength test was conducted according to the EN 1015-11:2001 standard (Figure 5).

**Figure 6:**
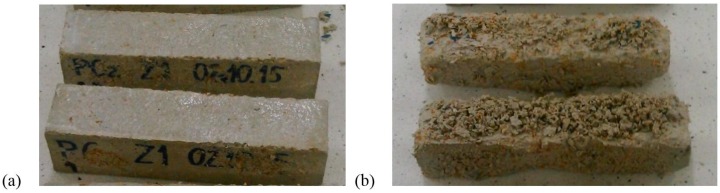
Samples of heat-insulating mortar after 50 freezing-thawing cycles: (**a**) mortar M1; (**b**) mortar M2.

Frost resistance of the studied mortars was determined using the direct method according to the EN 12012:2007 standard [36]. Samples with the dimensions of 40 × 40 × 160 mm were used. The mortars were tested in 50 cycles of freezing and thawing. Afterwards, the samples were dried to constant mass, and mass loss was determined. Figure 6 presents the destruction of the mortar with expanded cork aggregates after 50 freezing-thawing cycles.

Following the frost resistance test, the compressive strength test was conducted once more in order to determine the influence of the freezing-thawing cycles on the condition of the heat-insulating mortar samples.

**Figure 7:**
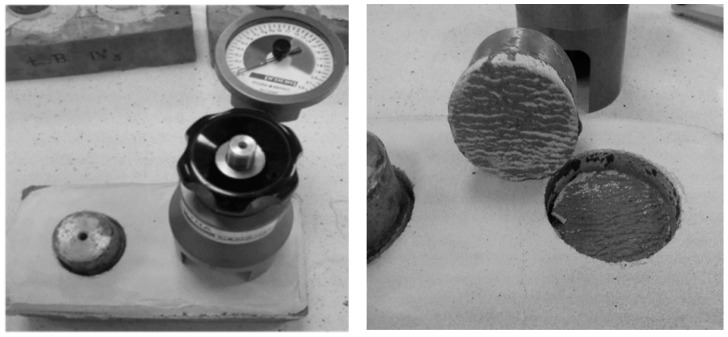
Determination of adhesive strength of mortar M2.

The determination of the adhesive strength of the tested mortars was performed directly according to the EN 1015-12 standard [37] with application of a pull-off tester TPO-W10A by Ar Ho, Poland (see Figure 7). The adhesive strength was determined as the maximal tensile stress caused by detaching load perpendicular to the surface of the mortar. The detaching load was introduced by a detaching plate glued to the surface of the tested mortar specimen. The class 10 ceramic brick was used as a base surface for the adhesive strength tests. The measurement procedure is presented in Figure 7. The applied pull-off tester with a test plate diameter equal to 50 mm allowed for ±1% accuracy for the whole range of the measured values.

Determination of surface roughness and three-dimensional (3D) topography was conducted in a T8000 RC120-400 device by JENOPTIK, Jena, Germany. The measurements were performed with the use of the unified GUI, allowing calculations of all parameters of tested roughness and waviness profiles as well as assessment of the geometrical characteristics, including distances, angles, maximal peaks, and valleys of the studied surface.

The morphology, porous structure of the mortars, and interfacial transition zone between the paste and the fine aggregate were determined with scanning electron microscopy SEM. Observations were performed by means of a Quanta FEG 250 microscope, by FEI, Hillsboro, OR, USA. Powdered samples for SEM observations were stuck to a carbon holder with carbon glue. Then they were dusted with an approx. 50-nm thick carbon layer in a sputter coater to achieve conductivity on the material surface.

## 3. Results and Discussion

The physical properties of the heat-insulating mortars adopted for the examination are shown in Table 5, while the mechanical properties are presented in Table 6.

On the basis of the previous reports [3,4], it can be assumed that as the amount of the lightweight fine aggregate, i.e., expanded cork, in the mortars increases, the mechanical properties, including compressive strength, flexural strength, and frost resistance, will decrease. However, the insulating parameters, porosity, and absorbability will increase. In the paper, the influence of binders such as cement, lime, or different fine aggregates on mortar properties is shown.

The heat-insulating mortars with cork produced in our study had a density of 2230–2490 kg∙m^−3^. The results show that the amount of expanded cork and hydrated lime, as well as the lack of sand, contributed to an increase in mortar absorptivity and a decrease in mortar density. The decrease in the density ranges from 17% to 36% and increases accordingly with the amount of hydrated lime and cork (added 25% and 20%, respectively), when compared to the samples without lime. The open porosity of the mortars was within the range of 24.66–48.68% and corresponded to values reported for the lightweight mortars. The addition of hydrated lime at a level of 25% and the lack of sand caused a 49.3% increase in open porosity and increased the absorptivity from 15.75 to 48.46%. The highest tightness (62.75%) was found for the M2 mortar (26.52%) without lime, but with the highest amount of cement and sand. When the amount of expanded cork was halved (M4), in comparison to the M1, there was an over 22% decrease in absorptivity. Table 5 shows the lowest thermal conductivity in the case of the M1 mortar, while the highest value was found in the M2 mortar with the highest addition of cement and sand. When the hydrated lime was used and when the cement amount was increased to the level of 5%, the thermal parameters decreased by approx. 35% (M1). Halving the cork amount caused an increase in the coefficient λ by approx. 18% (M4). The M3 mortar, which consisted of the same percent of components (20%), exhibited the most similar average results of the specific parameters.

Based on the results of the strength tests presented in Table 6, it can be seen that the addition of expanded cork ranged from 10 to 20% and the 5% reduction in the amount of cement and lime decreased the compressive strength by 49%. The final strength considerably increased by approx. 56% when the expanded cork additive was maintained at the level of 20% with the lime and the cement level increased to 5%. The key role in the strength increase was played by the increase in the cement content. A similar situation can be observed in the case of flexural strength results. The determined flexural strength of mortar M2 was almost two-fold higher than in mortar M1, which contained no sand. Relatively high strength was exhibited by the mortar with 10% content of expanded cork, but such a result was expected because the amount of the lightweight fine aggregate was two-fold lower. The presence of the acetate copolymer had an influence on the strength increase, which was also shown in studies on different lightweight mortars with lightweight expanded clay aggregate [6] and hemp composites [38]. During the frost corrosion test, significant differences in the behavior of the analyzed mortars were caused by the freezing and thawing cycles. M1 were the most damaged samples. The destruction of whole samples was noticed, including chipping (Figure 6b) and disintegration of samples. The M2 samples were more frost resistant than the other ones; they remained almost undamaged—there were neither cracks nor chipping on the surface of the rectangular prism (Figure 6a). The M4 samples, as well as the M2 samples, were not significantly damaged, but the scale of delamination was slightly greater in comparison to the M2 samples. After the frost resistance test, the delaminations and losses of the surface layer of mortar M3 were observed. After 50 cycles, the M1 sample showed the lowest frost resistance, i.e., 14.6% weight loss, which was by 94% higher than for the mortars with the two-fold higher content of expanded cork (M4).

All of the tested mortars exhibited high values of adhesion strength, in the range of 0.23–0.51 N∙mm^−2^, and the highest value was observed for the M2 mortar (see Table 6 and Figure 7), which was also characterized by the highest value of compressive strength. In this case, the adhesion strength of the mortar was over two-fold higher than that noted for the mortar containing sand. It was also observed that the increase in the adhesive strength of the mortars was related to an increase in its compressive strength. In the case of lightweight mortars with relatively low strength characteristics, in contrast to equivalent mortars without lightweight aggregates, the adhesion strength, directly related to their surface roughness, seems to be the key factor.

The adhesion of mortars depends not only on their morphology and porosity of the base surface, which seems to be obvious, but also on the roughness of the mortar itself, which was underlined in this study. Mortars containing a fine lightweight aggregate, such as expanded cork, have quite an uneven surface, which can be characterized by the following parameters: R_a_—Average Roughness defined as the average deviation of the profile in relation to its mean line and a parameter more sensitive to peaks and valleys R_q_—Root-Mean-Square Roughness. However, R_a_ and R_q_ do not provide the required information concerning the local variability of the surface profile. There is a possibility that two different profiles may be characterized by the same average values. Thus, alternative measures of roughness descriptions were proposed [39], taking into consideration the localization and distances between peaks and valleys. These include the Mean Peak Height (R_pm_) defined as the mean peak height from each length of sampling, the Mean Valley Depth (R_vm_) as the mean maximum value of the valley depth for each length of sampling, the Maximum Peak Height (R_p_) as the maximum height of the peak within the evaluation length, the Maximum Valley Depth (R_v_) as the maximum depth observed within the evaluation length, the Ten Points Height (R_z_) as the mean value of the sum of five the highest peaks with five lowest valleys for each line of evaluation, and, finally, the Maximum Peak-to-Valley Height (R_max_) defined as the maximum peak-to-valley height within any of the sampling lengths (R_max_ = R_v_ + R_p_).

The average characteristics of roughness obtained for the tested mortars are presented in Table 7.

The results of the studies of the roughness of cork-based mortars presented in Table 7 showed the diversification of the geometrical structure of their surface, taking into account the aspect of their mechanical adhesion to the surface, in this case to the ceramic brick.

The structure of mortars may affect their ability to penetrate the irregularities of the brick surface and may result in an increase in mechanical adhesion; thus, it may influence the strength of adhesive connections. Our studies showed that mortar M4, without sand and containing a two-fold lower amount of cork, exhibited the lowest characteristics of roughness, the Maximum Peak Height (R_p_), the Maximum Valley Depth (R_v_), as well as the Average Roughness (R_a_ = 24.2 µm). This mortar is also characterized by high strength parameters and high adhesion that is equal to 0.48 N∙mm^−2^ (Table 6), which may be caused by the low content of the lightweight aggregate and low roughness characteristics. Since this mortar is characterized by low values of R_vm_ and R_v_, describing the maximum depth of valley, we can conclude that it has the smoothest surface of all the other tested mortars. In our opinion, this feature determines the tight penetration of the M4 mortar into the rugged surface of the ceramic base, increasing the adhesive strength between the mortar and the brick base. On the other hand, the highest strength characteristics and parameters of roughness were observed for the M2 mortar containing sand and two-fold greater content of expanded cork. The determined Maximum Peak-to-Valley Height (R_max_) for M2 was by 26% higher than for mortar M4, as characterized by the lowest roughness.

Brás et al. [3] showed that using 50% of cork as a sand replacement causes no major differences in the compressive strength of mortars and the value is just 20% of the compressive strength of a conventional mortar. The reported mechanical strength tended to decrease when a greater cork dosage was used. The thermal conductivity determined by Brás A. et al. [3] was significantly higher than the value presented in this research, but this was caused by higher density and was equal to 1.5 W∙m^−1^∙K^−1^ and 0.5 W∙m^−1^∙K^−1^. The M2 and M3 mortars presented in this study were characterized by higher density: 1560 kg∙m^−3^ and 1420 kg∙m^−3^, for which the λ coefficients were 0.478 and 0.428 W∙m^−1^∙K^−1^, respectively.

While studying clay plasters with cork, Maaloufa Y. et al. [40] noticed that the thermal conductivity decreased from 0.51 for clay alone to 0.246 W∙m^−1^∙K^−1^ for the composites with 100% cork and that their density decreased as well. The Moreira’s [4] experimental results pertaining to the mechanical and hygrothermal characterization showed that replacing sand with expanded cork in the mixtures lowers their hardened density, compressive strength, and thermal conductivity. It was also shown that the mixtures with the lowest cement content have lower thermal conductivity. Similar results were observed in our research. In the research carried out by Panesar D.K. et al. [7], it was found that the range of strength in mixtures with 20% cork as sand replacement was much narrower, reaching 10.2–11.3 MPa after 56 days. The research also showed that the thermal conductivity of concrete composites with cork decreased as the concrete density dropped [7].

**Figure 8:**
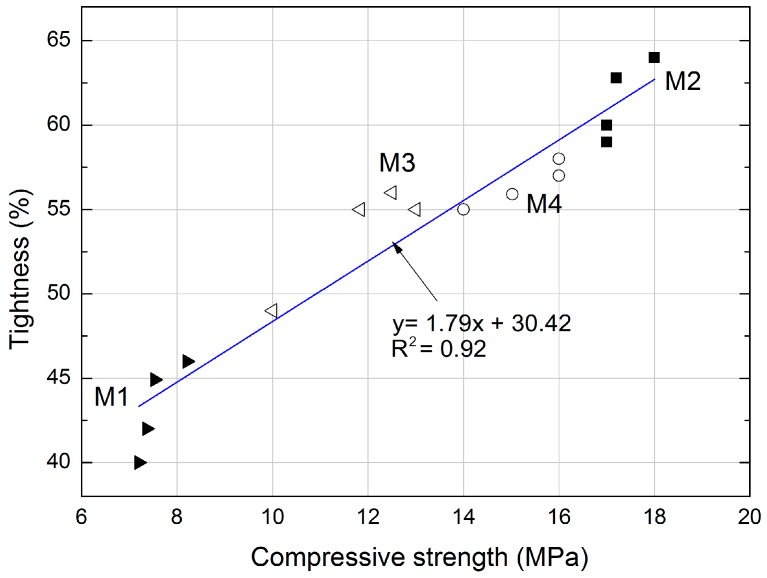
Correlation between compressive strength and tightness of mortars with expanded cork.

**Figure 9:**
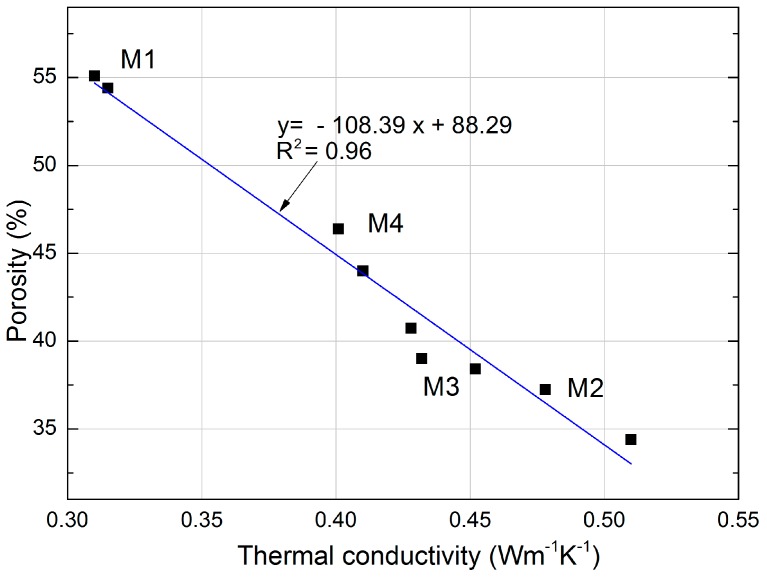
Correlation between thermal conductivity and porosity of mortars with expanded cork.

The graphs (Figure 8 and Figure 9) show the dependence of the different properties studied. The cube compressive strength on the tightness for the mortars is shown in Figure 8. In this study, the determined compressive strength corresponds directly to the tightness of mortars containing expanded cork. The linear trend was characterized by a good correlation coefficient R^2^ = 0.92 and relatively low errors in the intercept. The influence of tightness on compressive strength is shown in Figure 8. The high cement and sand content, combined with the reduced (halved) cork content, influenced the tightness in the M4 mortar.

A correlation between the thermal conductivity and porosity of mortars with expanded cork is shown in Figure 9. It can be noticed that there is a close correlation between porosity and the thermal conductivity coefficient of mortars with cork. The linear function shows a very good determination coefficient R^2^ equal to 0.96. Mortars containing no sand but hydrated lime showed significantly greater porosity than the other tested specimens, regardless of the applied amount of cork, 10% or 20% (mortars M1 and M2).

**Figure 10:**
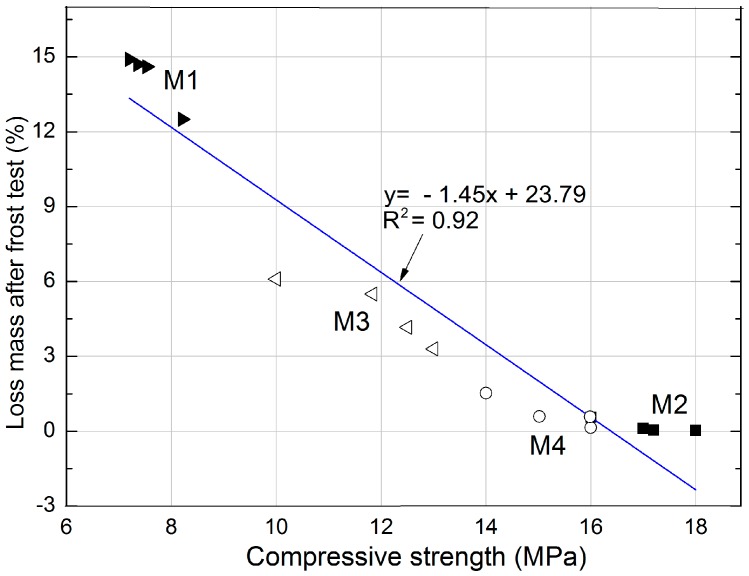
Correlation between compressive strength and frost resistance.

The following model (Figure 10) presents the extent to which the characteristic of mortar with expanded cork influences the frost resistance, which indirectly defines the corrosion resistance of the material. Figure 10 shows the linear correlation between frost resistance and compressive strength. These correlations can be described by the equation: y=−1.45x+23.79, which is characterized by a high coefficient of determination R^2^ = 0.92.

Based on Figure 10 and Table 6, it can be concluded that frost resistance is closely related to the compressive strength. The lower the strength, the lower the frost resistance and the greater the mass loss. The M2 mortar containing sand exhibits the highest determined compressive strength, which clearly corresponds to the lowest mass loss after the frost resistance test.

Knowledge of these dependencies can be useful not only in practice during the selection of the suitable mortar, but also can serve as a basis for designing the composition of heat-insulating mortars with expanded cork earmarked for facades exposed to frost.

**Figure 11:**
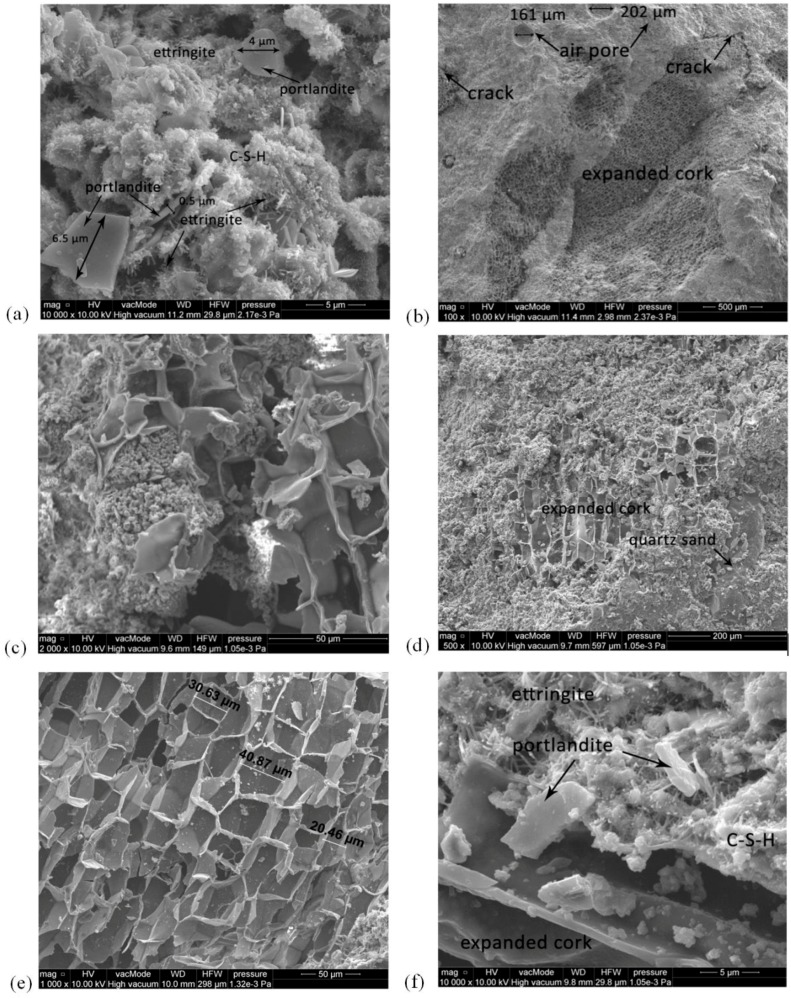
Microstructure of mortars: (**a**) M1—10,000×; (**b**) M1—100×, (**c**) M3—2000×, (**d**) M3—500×, (**e**) M1—1000×; (**f**) M3—10,000×.

The microstructure of the studied mortars containing cork as filler (M1, M3) is shown in Figure 11. Figure 11a,f (10,000×) show the microstructure of mortars M1 and M3, rich in portlandite and ettringite particles. There is visible amorphous hydrated gel of the C-S-H phase. The interfacial transition zone between the cement-lime paste and expanded cork aggregates and the structure of cork containing many micro polyhedrons (Figure 11b,e) are also shown. The mortar exhibited mostly very good bonding with the cork aggregate; however, there were micro-cracks visible in the cement paste with the mean width of approx. 20 µm (Figure 11a). The M1 mortar structure contains a few air voids, ranging from 160 to 200 μm. Air voids and cracks between expanded cork and cement paste could result in changes in the typical characteristics of the analyzed mortar, including an increase in porosity and a decrease in the thermal conductivity coefficient, strength, and frost resistance of the tested specimens.

The high level of roughness and good adhesion between the cork aggregate or quartz sand and the cement-lime paste in M3 guaranteeing high durability are confirmed in Figure 11c,d. The scanning electron microscopy pictures showed neither cracks nor air voids that could cause a decrease in the porosity and absorbability of M3.

## 4. Conclusions

The following key conclusions can be drawn from this study:

- The strength of adhesive bonds between the mortar and the brick base is influenced not only by roughness of mortars but also by the composition of the mortar itself. The highest strength was noted for the M2 mortar, which was also characterized by the highest parameters of roughness. The Average Roughness of the M2 mortar was 33% higher than R_a_ of by M2 mortar, which had the lowest roughness.
- The high roughness of the M2 mortar limits tight penetration of the mortar into the surface of the ceramic base material, which may negatively influence the strength and durability of bonding between the mortar and the base material. However, our studies showed that, despite the high roughness, the M2 mortar showed the greatest adhesive strength, which may be related to the composition of the mortar. M2 contains the highest amount of Portland cement and silica derived from quartz sand, significantly increasing its strength parameters.
- The high values of R_p_ and R_v_ may also trigger non-uniform adherence of the mortar to the ceramic base, which may lead to penetration of microcracks and microvoids by water, resulting in the low frost resistance of the M1 and M3 mortars. On the other hand, despite the highest values of R_p_ and R_v_, the determined frost resistance of the M2 mortar was the highest, due to the application of the greatest amounts of cement and sand.
- Despite the best strength characteristics, the M2 mortar does not comply with the requirements for heat-insulating mortars due to the very high coefficient of thermal conductivity of almost approx. 0.50 W∙m^−1^∙K^−1^. The analysis of the results indicates that when sand is replaced by expanded cork, the density, compressive strength, and thermal conductivity of the mortar decrease. Moreover, the tests show that *Quercus suber* oak cork has a positive influence on increasing the thermal conductivity of mortars.
- Very good linear correlations between the thermal conductivity coefficient and the porosity of the mortars, as well as between compressive strength and tightness were obtained. Based on the conducted study, the M1 mortar achieved the lowest thermal conductivity coefficient of 0.310 W∙m^−1^∙K^−1^, while absorptivity was 48.46%, porosity was 55.09%, and the loss of mass after the frost resistance test reached a high value of approx. 14.6%. This makes it a suitable heat-insulating mortar but rather inappropriate as a durable construction mortar.
- The highest tightness of 62.75% was exhibited by the M2 mortar without lime, but with the highest amount of cement and sand.
- The flexural strength of the M2 mortar was almost two-fold higher (4.80 MPa) and its compressive strength was by approx. 56% higher than in mortar M1, which contained no sand. The M2 mortar containing no lime also achieved the best frost resistance (0.1% loss mass). The silica content significantly increased the flexural and compressive strength as well as the frost resistance.
- The 50% lower amount of expanded cork used instead of sand resulted in an approx. 18% higher observed value of the thermal conductivity coefficient of the mortar.
- The studied mortars exhibited very good bonding with the cork aggregate; however, there were micro-cracks visible in the cement paste of M1 with the mean width of approx. 20 µm, which was determined during the SEM examination.
- It was shown in this paper that the microstructure and roughness of mortars can be used to predict their strength and adhesion to the base. The M2 mortar, different in the composition from the other specimens, was the only exception. The high content of cement and sand significantly improved the strength characteristics, despite the greatest roughness. In the case of the other mortars, an increase in the roughness and porosity was followed by a decrease in their coating and adhesive strength. It should be underlined that different relations were observed during our studies of the mortars than for mineral bases. As already reported [41,42,43], the high roughness and the porosity of the base ensure a good coating.

## Figures and Tables

**Table 1:** Composition of the mortars with expanded cork aggregate.

Materials	Unit	M1	M2	M3	M4
Portland cement I 32.5R	(vol %)	25	30	20	30
Expanded cork 0.5–2 mm	(vol %)	20	20	20	10
Hydrated lime	(vol %)	25	-	20	30
Quartz sand 0.075–2 mm	(vol %)	-	30	20	-
Water	(vol %)	29.85	19.85	20	29.85
Ethylene vinyl acetate copolymer	(vol %)	0.15	0.15	-	0.15

**Table 2:** Technical parameters of Portland cement CEM I 32.5R.

Parameters	Unit	Value
Specific surface	(cm^2^∙g^−1^)	3985
Initial setting time	(min)	248
Compressive strength after 2 days after 28 days	(MPa)	17.6 43.2
Loss on ignition by cement weight	(%)	5.0
Density	(g∙cm^−3^)	3.05
Volume stability	(mm)	<10
SO_3_ content	(%)	2.798
Cl content	(%)	0.066
Cr(VI) diss. content	(ppm)	0.26
Na_2_O_eq_ content	(%)	0.78

**Table 3:** Chemical composition of quartz sand.

Composition	Unit	Value
SiO_2_	(vol %)	95.3
Al_2_O_3_	(vol %)	1.9
Fe_2_O_3_	(vol %)	0.7
CaO	(vol %)	0.35

**Table 4:** Parameters and components of expanded cork.

Parameters	Unit	Value
Density of fraction 0.5–1 mm of fraction 1–2 mm	(kg∙m^−3^)	55–65 65–75
Humidity	(%)	max 6
Thermal conductivity coefficient of fraction 0.5–1 mm of fraction 1–2 mm	(W∙m^−1^∙K^−1^)	0.04 0.036
Absorbability	(kg∙m^−3^)	0.5
Fire resistance rating	(°C)	Euroclass E from −180 to 120
Components		
suberin	(mass %)	45
lignin	(mass %)	25
cellulose	(mass %)	13
extractables	(mass %)	10
ash	(mass %)	2
others	(mass %)	5

**Table 5:** Physical properties of heat-insulating mortars with expanded cork.

Type of Mortars/Descriptive Statistics		Apparent Density (kg∙m^−3^)	Density (kg∙m^−3^)	Open Porosity (%)	Tightness (%)	Total Porosity (%)	Absorptivity (%)	Thermal Conductivity Coefficient (W∙m^−1^∙K^−1^)
M1	Mean	2230	1000	48.68	44.91	55.09	48.46	0.310
	SD	0.22	0.01	2.31	3.42	3.42	2.61	0.02
	CV	0.45	0.68	4.75	6.54	6.54	5.39	6.40
M2	Mean	2490	1560	24.66	62.75	37.25	15.75	0.478
	SD	0.32	0.01	0.93	2.87	2.87	0.53	0.003
	CV	0.21	0.54	3.75	4.43	4.43	3.38	0.15
M3	Mean	2400	1420	33.47	59.27	40.73	23.48	0.428
	SD	0.05	0.01	1.06	5.01	5.01	0.67	0.02
	CV	0.66	0.48	3.18	1.98	1.98	2.85	4.21
M4	Mean	2270	1200	45.42	52.93	47.07	37.79	0.378
	SD	0.21	0.01	1.23	2.19	2.19	0.93	0.006
	CV	0.98	0.35	2.71	1.01	1.01	2.47	0.63

Coefficient of variation—CV, Standard deviation—SD.

**Table 6:** Mechanical properties of heat-insulating mortars with expanded cork.

Type of Mortars/Descriptive Statistics		Compressive Strength (MPa)	Flexural Tensile Strength (MPa)	Frost Resistance * (%)	Adhesion (N∙mm^−^^2^)	Loss (+)/Increase (−) of Compressive Strength after Frost Test (%)
M1	Mean	7.53	2.53	14.6	0.23	-
	SD	0.24	0.16	1.0	0.33	-
	CV	3.18	6.26	1.7	1.34	-
M2	Mean	17.20	4.80	0.1	0.51	−23.15
	SD	0.45	0.22	0.1	0.10	0.87
	CV	2.61	4.48	11.7	1.45	6.65
M3	Mean	11.84	2.92	5.5	0.41	-
	SD	0.38	0.20	0.2	0.09	-
	CV	3.18	6.90	34.8	1.98	-
M4	Mean	15.02	3.84	0.9	0.48	14.65
	SD	0.31	0.06	5.6	0.09	0.57
	CV	2.09	1.55	24.9	2.01	8.14

* Loss of mass after testing frost resistance, - no data.

**Table 7:** Characteristics of roughness for all tested cork-containing mortars.

Type of Mortars	Mortar Roughness Characteristics (µm)							
	R_a_	R_q_	R_vm_	R_pm_	R_p_	R_v_	R_z_	R_max_
M1	29.2	33.5	190	78	89	229	274	318
M2	36.1	39.9	203	96	102	281	331	383
M3	30.4	30.1	199	81	94	246	298	340
M4	24.2	26	153	72	81	201	231	282
